# Drug-coated balloon for treatment of non-atherosclerotic renal artery stenosis—a multi-center study

**DOI:** 10.1186/s12872-023-03484-5

**Published:** 2023-10-16

**Authors:** Xitao Song, Yining Fu, Zhichao Lai, Xiao Di, Rong Zeng, Jiang Shao, Leng Ni, Zhili Liu, Xiaojun Song, Wei Ye, Changwei Liu, Bao Liu, Yuehong Zheng, Yuexin Chen

**Affiliations:** Department of Vascular Surgery, Peking Union Medical College Hospital, Chinese Academy of Medical Sciences, Shuaifuyuan 1st, Dongcheng District, Beijing, 100730 China

**Keywords:** Hypertension, Renal artery obstruction, Angioplasty, Balloon, Treatment outcome, Research design

## Abstract

**Introduction:**

Renal artery stenosis (RAS) is a significant reason for secondary hypertension. Impaired renal function and subsequent cardiopulmonary dysfunction could also occur. Patients of non-atherosclerotic RAS has a relatively young age and long life expectancy. Revascularization with percutaneous transluminal angioplasty (PTA) is a viable treatment option. However, restenosis is unavoidable which limits its use. Drug-coated balloon (DCB) has been proven to be effective in restenosis prevention in femoropopliteal arterial diseases and in patients with renal artery stenosis. And PTA for Renal artery fibromuscular dysplasia is safe and clinically successful. Therefore, we could speculate that DCB might have potential efficacy in non-atherosclerotic RAS treatment.

**Methods and analysis:**

This will be a randomized multi-center-controlled trial. Eighty-four eligible participants will be assigned randomly in a 1:1 ratio to the control group (plain old balloon, POB) and the experimental group (DCB). Subjects in the former group will receive balloon dilatation alone, and in the latter group will undergo the DCB angioplasty. The DCB used in this study will be a paclitaxel-coated balloon (Orchid, Acotec Scientific Holdings Limited, Beijing, China). Follow-up visits will be scheduled 1, 3, 6, 9, and 12 months after the intervention. Primary outcomes will include controlled blood pressure and primary patency in the 9-month follow-up. Secondary outcomes will include technical success rate, complication rate, and bail-out stenting rate.

**Trial registration:**

ClinicalTrials.gov (number NCT 05858190).

Protocol version V.4 (3 May 2023).

**Supplementary Information:**

The online version contains supplementary material available at 10.1186/s12872-023-03484-5.

## Strengths and limitations

- ► Non-atherosclerotic renal artery stenosis accounts for 10% of hypertension in young patients. With long life expectancy and severe consequences, these patients deserved enough attention and timely treatment.
- ► The effectiveness of the drug-coated balloon (DCB) was verified in patients with lower extremity arteriosclerosis, however, for patients with renal artery stenosis, existing studies were mainly retrospective studies, which means high-quality studies are still needed.
- ► The DCB showed strengths compared with open surgery, stent implantation, and plain old balloon (POB) since the balloon dilation was less traumatic and free of stent-related complications. Furthermore, the loaded drug may reduce the restenosis rate by inhibiting smooth muscle proliferation.
- ► As a randomized controlled trial, all participants are blind to the group assignment, which may reduce the expectancy-induced bias.
- ► CTA or DSA will be performed under the regular follow-up to evaluate the patency rate. Regular follow-up leads to timely communication and an adequate understanding of the disease.
- ► Different from open surgery, balloon dilatation may need repeated procedures, leading to vascular complications.

## Introduction

Renal artery stenosis (RAS) is acommon vascular disease usually caused by atherosclerosis. It can also be due to non-atherosclerotic reasons, such as fibromuscular dysplasia (FMD), Takayasu’s arteritis (TA), and neurofibromatosis [1]. RAS can lead to renovascular hypertension (RVH), renal insufficiency, and other secondary organ damage. RVH is the most common manifestation. RVH accounts for approximately 1% of hypertension in adults [2]. However, this number can increase to 10% in adolescent or pediatric patients [3]. RAS can also lead to renal deficiency and cardiac disturbance, particularly in patients with RAS on both sides or on a solitary kidney.

Renovascular revascularization, such as percutaneous transluminal angioplasty (PTA), stenting, bypass, and renal auto-transplantation, is a proven technique for treating RAS. PTA with or without stenting is the first-line choice in most cases as it is minimally invasive [4]. However, in patients with atherosclerotic RAS, stenting does not claim superiority to medical therapy for the long-term prevention of adverse cardiovascular events, salvage of renal function, and control of RVH [5, 6]. Its effect may differ in patients with non-atherosclerotic RAS. A meta-analysis reported that the combined rates of hypertension cured by angioplasty or surgery were estimated to be 46% (95% confidence interval [CI]: 40%–52%) and 58% (95% CI: 53%–62%) in patients with FMD [7].

Restenosis limits the use of PTA as the first choice of treatment for renovascular revascularization. Zhu et al*.*found that the restenosis rate was 40.9% in patients with TA after endovascular treatment [8]. For FMD, the rate for repeated PTA was 18.2% [95% CI: 11.0%–26.8%] in a meta-analysis [7]. The drug-coated balloon (DCB) has been designed to prevent restenosis by delivering antiproliferative drugs to the artery wall after PTA. It has been proven to be superior to plain old balloons (POBs) in the prevention of restenosis in patients with femoropopliteal artery disease [9, 10]. It has also shown satisfactory preliminary results in patients with non-atherosclerotic RAS [11, 12]. However, thus far, no randomized controlled trial has reported any strong evidence to support these results.

We speculate that the paclitaxel-eluting balloon would have potential efficacy in patients with non-atherosclerotic RAS. However, this theory needs to be tested using randomized control trials (RCTs). Therefore, we designed this RCT to evaluate the safety and efficacy of DCB compared with POB.

### Objective

This study aims to evaluate the efficacy of DCB compared with POB in patients with non-atherosclerotic RAS through a RCT.

## Methods and analysis

### Study design

**Fig. 1:**
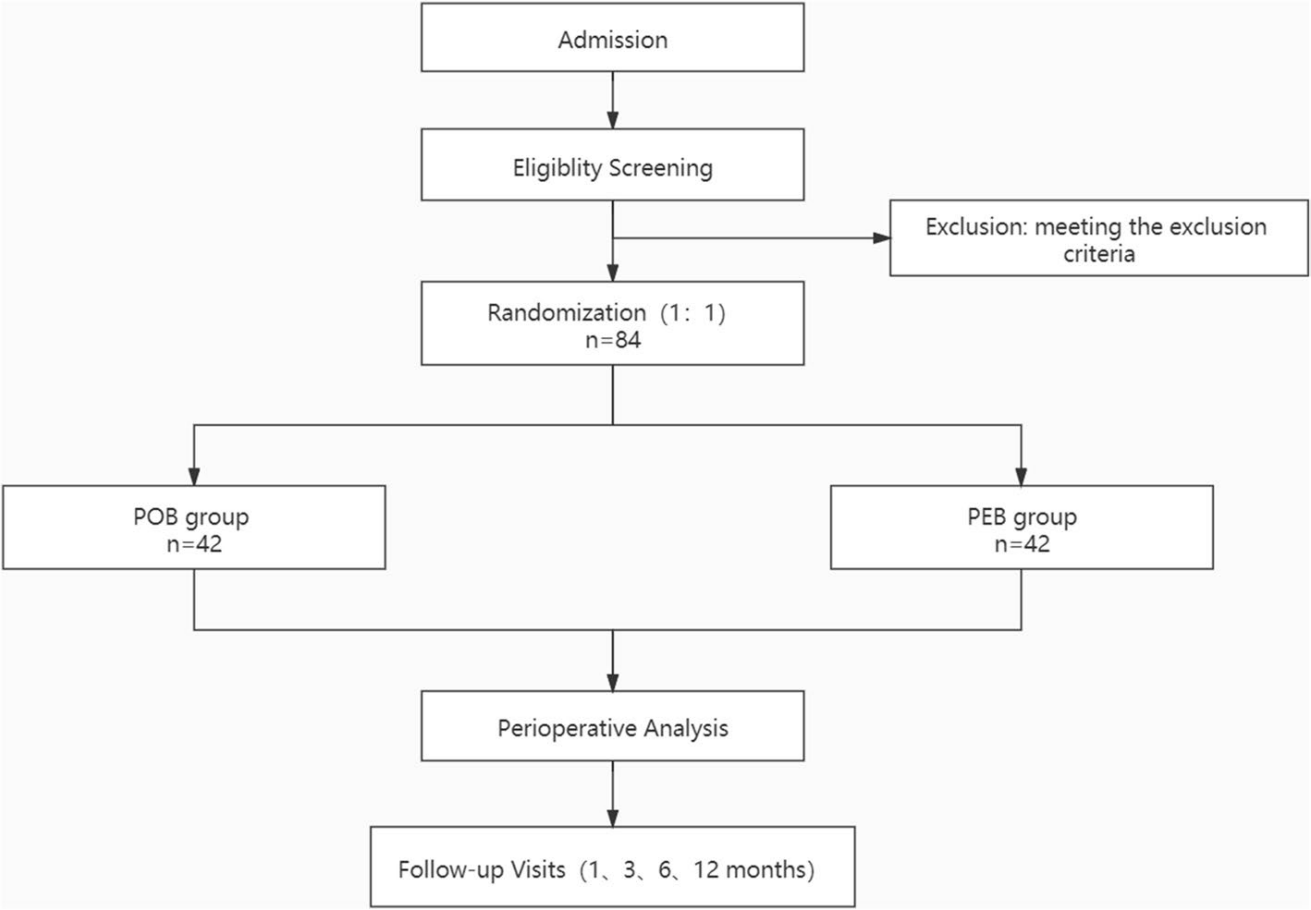
Gives a brief introduction of the study flow

This study is a superiority trial. As a multi-center, randomized controlled study, this study will compare DCB with POB for angioplasty in patients with non-atherosclerotic RAS. We will allocate eligible participants through a computer-generated randomization scheme. Patients, study personnel, and statisticians will remain blind to the assignment. However, the operator will know the assignment as the DCB looks different from POB. The trial has been registered with ClinicalTrials.gov (number NCT 05858190). The protocol has been approved by the Ethics Committee of PUMCH (number: HS-2133). An independent data and safety monitoring committee will review the efficacy of this trial. All participants will provide written informed consent before enrollment. Figure 1 presents a brief introduction of the study flow.

### Study setting

The setting for this study is in Beijing, China. The study had a multi-center design in public academic hospitals.

### Eligibility criteria

#### Inclusion criteria

Participants who meet the following criteria will be qualified:

- ➣ 18–45 years of age.
- ➣ Unilateral or bilateral RAS with stenosis diameter ≥ 60% on computer tomography angiography (CTA), digital subtraction angiography (DSA), or magnetic resonance angiography (MRA). The calculation formula for the stenosis degree is [(proximal or distal) normal vessel diameter − residual diameter at the stenosis site]/(proximal or distal) normal vessel diameter > 100% [13].
- ➣ Hypertension (SBP ≥ 140 mmHg and/or DBP ≥ 90 mmHg). The blood pressure will be measured according to the method recommended by the American College of Cardiology/American Heart Association (ACC/AHA) in 2017 [14].
- ➣ With no severe renal insufficiency: length of the targeted kidney ≤ 7 cm; estimated glomerular filtration rate (eGFR) < 30 mL/min.
- Furthermore, eGFR will be calculated according to the Cockcroft and Gault formula as follows: GFR (mL/min) = [(140 − age) × weight × (0.85 female)]/[72 × Scr (mg/dL)] [15].
- ➣ Having good compliance with the study protocol.
- ➣ Giving informed consent.

#### Exclusion criteria

Participants who meet the following criteria will be excluded:

- ➣ With apparent atherosclerotic risk factors.
- ➣ History of renal intervention or surgery.
- ➣ With congenital anatomical anomaly.

With severe renal insufficiency (length of the target kidney < 7 cm, eGFR < 30 mL/min; divided eGFR of the target kidney < 8 mL/min) [16].

The divided eGFR of the target kidney will be measured by renal dynamic imaging.

- ➣ With contraindication for antiplatelet therapy.
- ➣ With severe cardiopulmonary complications.
- ➣ Known allergy to contrast media.
- ➣ Pregnancy or preparing for pregnancy.
- ➣ With active cancer.
- ➣ Life expectancy < 12 months.
- ➣ Reluctance to provide informed consent.

#### Dropout criteria

Enrolled participants who meet the following criteria shall be considered dropout cases:

- ➣ Subjects with poor compliance caused by themselves or medical reasons.
- ➣ Loss of crucial records due to various reasons.
- ➣ Withdrawal of informed consent.
- ➣ Abandoning the study (including abandonment due to ineffectiveness of the intervention).
- ➣ Worsening of pre-existing underlying diseases or occurrence of other severe adverse events due to non-intervention reasons.
- ➣ Removal of patients who need rescue therapy with the stent as dropout cases, as their existence may bias the final results. Post this process, we will figure out the reasons for the rescue stent and record the stent brand and model in detail. Finally, we will count the proportion requiring the rescue stents in the POB arm and the DCB arm as a secondary outcome measure and reflect it objectively in the final results.

### Interventions

#### POB arm

POB, that is the commercially available, non-compliant balloon, will be selected according to the operators’ preference. Angiography will be performed via the transfemoral artery or brachial artery approach to the upper abdominal aorta in order to identify the site and degree of the RAS. Then, a catheter will be advanced into the targeted renal artery for the actual evaluation and location of the lesion. Thereafter, POB will be introduced to the predetermined location and dilatated. Further procedures will depend on the outcomes of the PTA results. If the PTA can provide sufficient dilation with residual stenosis ≤ 30%, it can serve as a beneficial therapy. However, if the residual stenosis is greater than 30% or flow-limiting dissection occurs, bare-metal stents will be implanted.

#### DCB arm

The DCB used is a paclitaxel-coated balloon (Orchid, Acotec Scientific Holdings Limited, Beijing, China). The operator will first perform PTA with POB in the same manner as in the POB arm. A DCB with the same diameter as that of the previous POB will then be introduced to the target vessel area and dilated. The dilatation will last for 3 min for depositing paclitaxel into the arterial wall. Thereafter, the final angiography will be performed to evaluate the vessel lumen.

Medicine interventions (e.g.immune-suppressing medication to control the progress of TA) are permitted during the trial.

### Endpoints

#### Primary endpoints

Primary endpoints are the clinical benefits and the primary patency rate at the 9-month visit.

The clinical benefits refer to controlled blood pressure (cure or improvement) following the evaluation criteria recommended by Rundback et al [13]. “Cure” means the subjects’ blood pressure is within 95% CI of the population at the same age under the condition of not taking any anti-hypertensive drugs. “Improvement” suggests the dosage or number of tablets of the medicine decreases or the blood pressure decreases while it is still higher than the normal parameters. “Failure” implies no significant improvement in blood pressure or no dosage of medicine reduction.

Primary patency rate is defined as no restenosis occurring with the luminal diameter of the blood vessel being less than 50%, as confirmed by CTA or DSA during the follow-up. Because of the potential side effects (X-ray exposure, contrast nephropathy, intervention-related complications, etc.), CTA or DSA will be performed 9 months after the index procedure in most cases. However, whenever clinical manifestations or ultrasonography implies restenosis, CTA or DSA should be processed. Color Doppler ultrasonography can be conducted every 3 months to evaluate the patency of the target artery. If the patients’ symptoms deteriorate or blood pressure increases again, renal artery color Doppler ultrasonography will be performed as soon as possible. When we suspect RAS (major indicators: renal artery peak systolic velocity (PSV) > 180 cm/s and renal artery/aorta PSV > 3.5/1), CTA or DSA will be used to establish the diagnosis.

#### Secondary endpoints

Secondary endpoints include technical success rate, complication rate, bail-out stenting rate, secondary patency rates, target lesion revascularization rates (TLR), and changes in the renal function. Technical success is defined as no significant elastic recoil, flow-limiting dissection, or residual stenosis of ≥ 30%. Postoperative complications are defined as any procedure-related adverse events occurring within 30 days, mainly including but not limited to death, myocardial infarction, cerebral infarction, cardiac insufficiency, arrhythmia, cerebral hemorrhage, acute pulmonary edema, renal failure, deterioration of renal function (Cr increase ≥ 50%), nephrectomy, renal infarction, renal artery thrombosis, renal artery rupture, renal parenchymal hemorrhage, embolism of other organs, severe complications at the puncture site (massive hemorrhage or hematoma requiring surgical treatment, pseudo-aneurysm, or arteriovenous fistula) and minor complications at the puncture site (little hemorrhage or hematoma, cutaneous nerve injury, etc.). Secondary patency means no restenosis (≥ 50%) occurs after repeated PTA for the treatment of restenosis. Target lesion revascularization (TLR) means performing repeated endovascular treatment or surgical intervention after restenosis.

### Sample size

For evaluating the sample size, we took the primary outcomes into account on the basis of historical studies and experience [17]. We expect to reduce the restenosis rate from approximately 50% to 15% in 9 months after the clinical intervention. With two-sided α = 0.05, β = 0.10, the targeted sample size in each group was approximately 35 patients. Thus, we increased it by 20% to 42 cases in case of dropouts due to multiple reasons.

### Allocation and blinding

Patients meeting the selection criteria will be assigned randomly to either of the groups with no awareness of the randomization. Because the number list will be generated with SPSS, investigators and outcome assessors will have no information other than the number of patients in each group. The random number sequence was sealed in opaque envelopes to conceal the allocation sequence until the interventions were assigned. The researchers will enroll participants and assign participants to interventions. However, the operators performing the PTA will inevitably know the allocation due to differences in the appearance and the operating procedures of the balloons. Throughout the trial, unblinding is not permitted. When statistics had to be performed, the data analyst was unblinded.

### Data management

#### Case report form (CRF)

The eligible participants will be matched with their CRFs written by the clinicians who will be unknown to the specific groups. CRFs will be triplicated, ensuring that the original records do not get copied mistakenly. The investigators will record situations such as adverse events and dropout cases objectively. The CRFs will not be allowed to be modified after being reviewed by the monitors.

### Data entry and modification

The database administrators of the statistical unit will be in charge of the data entry and management. Microsoft Excel will be used for data entry, and quality control will be performed by double entry and validation. Doubts in CRFs will be raised and figured out by asking the investigators through the question–answer table (DRO). The database administrators will modify, confirm, and enter the data on the basis of the DRO returned by the investigators.

### Data locking

Other than for fixing problems in the protocol, the data files will be locked after correction(s), which means the investigators, sponsors, and statistical analysis personnel will have no rights to make changes in them.

### Data retention

The institution will store the information present in the original data for at least two years. However, sponsors will have to do so for at least five years.

### Statistical analyses

All variables associated with efficacy and safety will be analyzed using different follow-up periods, and the analysis sets (intention-to-treat and per-protocol analysis) will be compared between the two groups, including blood pressure control rate, primary patency rate, renal function, TLR, secondary patency rate, technical success rate, 30-day clinical success rate, and incidence rate of complications. The analysis sets will include a full analysis set (FAS), per-protocol set (PP), and safety analysis set. Those who do not adhere to randomization will be analyzed as intention-to-treat.

### Analysis methods

The covariates and drop-out cases will be considered during the descriptive analysis, hypothesis test, and parameter estimation. The differences between groups will be evaluated using the chi-square test for binary variables, *t*-test for continuous variables with a normal distribution, and Mann–Whitney U test for variables that do not meet the normal distribution. Kaplan–Meier curves will be drawn for the survival analysis. The differences will be found by the statistical significance if the *p*-value is less than 0.05.

### Full analysis set (FAS)

The FAS can be obtained by minimally and fair eliminating data from all qualified subjects, which is as close to the ideal situation as possible according to the intention-to-treat (ITT) principle.

### Per protocol set (PPS)

As a subset of FAS, PPS includes the enrolled subjects who complete the whole process according to the protocol, except for those who terminate treatments or violate the protocol at least once. The general definition of a violation is a failure to meet the inclusion criteria, to take the drug according to the protocol, to report for visits, and so on. However, an accurate definition can only be formulated after the review.

### Safety analysis set (SS)

SS refers to the dataset of subjects who receive the medical therapy at least once.

### Data monitoring

We will perform the study in a sound environment to isolate external interference. There was no patient and public involvement in this research. Subjects will need to fully understand the trial’s significance and actively cooperate with the physicians. Designers and investigators will be professionally qualified. All the investigators will be familiarized with the methods, procedures, and different requirements of the project before participating in the study. Investigators will ensure that the contents of the CRF are detailed and reliable. The monitors will be responsible for regular sample inspections. At least 10% of the CRFs and the original records will be randomly inspected, including the patients’ general information, treatments, and test items. The preservation of archives, data processing, and calibration of relevant detection instruments will be conducted by qualified personnel.

### Trial termination

Termination refers to a scenario wherein the trial has to be stopped halfway. Before the termination, relevant parties (Institutional Review Board, Acotec Scientific Holdings Limited Company, and Chinese Food and Drug Administration) have to be informed in advance. The detailed termination criteria include the following:

- ➣ A severe adverse event occurs, irrespective of whether it is related to the investigational device.
- ➣ The investigator will end the trial in the best interests of the patient if the ineffective treatments aggravate the condition.
- ➣ Errors exist in the development or implementation of the protocol, so it is difficult to evaluate the effects.
- ➣ There is a severe protocol violation.
- ➣ Sponsors request termination.
- ➣ The patients or their guardians propose the withdrawal.

If patients abandon the trial, the physicians are supposed to fill in the reason and the date on the Case Report Form (CRF). If patients terminate the study because of adverse events, a close follow-up will be performed until the situation improves.

### Adverse events

Adverse events refer to a series of expected or unexpected symptoms, signs, diseases, or injuries, which may be temporally associated with the drug or device, but not causally related. Adverse events can be divided into three levels (mild, moderate, and severe).

“Mild” suggests that the pain is tolerable, no treatment is needed, and no side effects occur upon the management and the rehabilitation.

“Moderate” indicates that the pain is intolerable to the patients, requiring withdrawal or special treatment, thus affecting the prognosis.

“Severe” refers to the occurrence of events leading to death or permanent disability, needing immediate drug withdrawal or clinical intervention.

We will carry out a close observation and follow-up, keeping an eye on adverse events at the time of the procedure and during the follow-up visits. We will make every effort to maximize patients’ interests, including timely summarizing and providing feedback on the events, giving remedial therapy, or even terminating the trial if necessary.

### Ethics approval

We will conduct the trial in conformance to the Declaration of Helsinki (2000), Chinese Good Clinical Practice (GCP), and the related regulations. The enrollment patients will strictly follow the inclusion criteria and sign the written informed consent. A close follow-up will be performed to guarantee efficacy and safety. Every patient is eligible for cash grants during the follow-up. This protocol will be available only after the approval of the Ethics Committee, which will conduct an independent, fair, and timely review of the project submitted by the applicant. If any modification is needed in the study, it will be negotiated and signed by the investigators and the sponsors. The amendment will be reported to the Ethics Committee for approval before implementation. In addition to reviewing and supervising all the drug clinical trial projects undertaken in this institution, the Ethics Committee may take responsibility for clinical trials conducted by other institutions.

## Discussion

The study will aim to compare the treatment efficacy of POB and DCB of non-atherosclerotic RAS through a randomized controlled clinical trial. The experimental group will use DCB (a paclitaxel-eluting balloon), while the control group will only use POB. The POB mainly increases the lumen diameter through mechanical dilatation. The DCB coated with a layer of the mixture containing paclitaxel is supposed to prevent subsequent restenosis by inhibiting neointimal hyperplasia.

Young patients or children with a relatively long life expectancy usually encounter non-atherosclerotic RAS. On the one hand, long-term damage secondary to RVH might be more common in these patients than in older patients with atherosclerotic RAS. Some patients may even need to remove the involved kidney [18]. PTA in patients with non-atherosclerotic RAS works in long-term complication prevention in theory. On the other hand, insufficient compliance or medication intolerance are more common in young patients [19]. Renovascular revascularization also edges on less medication or fewer subsequent adverse effects.

As the life expectancy of the abovementioned patients is relatively long and some patients may have underlying diseases, such as TA, the postulated restenosis rate of PTA is high. Therefore, the prevention of restenosis after PTA is essential for patients with non-atherosclerotic RAS. Drug-eluting stent (DES) or balloon (DCB) can inhibit the proliferation of smooth muscle cells by locally delivering the anti-proliferative drugs and then preventing restenosis. DES was found to be superior to bare-metal stents in terms of lowering the restenosis rate in atherosclerotic RAS (7.2% *vs.*18.6%, *p* = 0.031) [20]. However, the permanent existence of the stent after the implantation of DES in a young patient may lead to restenosis due to mechanical stimulation and complications of stent displacement or fracture in the long run.

“Leaving nothing behind” represents the well-known strength of the drug-coated balloon. The treatment of lower extremity arterial disease with DCB has gained widespread use [21]. For RAS, the use of DCB has been reported tentatively. It was first reported in 2013 by Itani et al*.*, who successfully used a DCB to treat a renal artery in-stent restenosis [11]. Then, Hecht et al*.*reported a supportive result of DCB in a patient with TA-related RAS in 2015 [12]. In 2018, Li et al*.*reported a large case series of 14 cases with RAS after renal transplantation who were treated with the paclitaxel-eluting balloon angioplasty. The technical success rate was 100%, and in a median 8-month follow-up, no reintervention was required [22]. The abovementioned results were delightful though the evidence level was relatively low.

In conclusion, a paclitaxel-eluting balloon may be theoretically effective in patients with non-atherosclerotic RAS. High-level evidence is needed to confirm this idea. Therefore, we will conduct this RCT to evaluate the efficacy of DCB in such patients.

### Supplementary Information

**Additional file 1.**

## Data Availability

The datasets used and/or analyzed during the current study available from the corresponding author on reasonable request.
